# Potential factors associated with clinical stage of nasopharyngeal carcinoma at diagnosis: a case–control study

**DOI:** 10.1186/s40880-017-0239-y

**Published:** 2017-09-04

**Authors:** Jun-Ting Ren, Meng-Yu Li, Xiao-Wen Wang, Wen-Qiong Xue, Ze-Fang Ren, Wei-Hua Jia

**Affiliations:** 1 School of Public Health, Sun Yat-sen University, 74 Zhongshan 2nd Rd, Guangzhou, 510080 Guangdong P. R. China; 2 State Key Laboratory of Oncology in South China, Collaborative Innovation Center for Cancer Medicine, Sun Yat-sen University Cancer Center, 651 Dongfeng East Rd, Guangzhou, 510060 Guangdong P. R. China

**Keywords:** Nasopharyngeal carcinoma, Stage, Socioeconomic status, Cancer cognition, China

## Abstract

**Background:**

In China, most patients with nasopharyngeal carcinoma (NPC) are diagnosed at a late stage and consequently have a poor prognosis. This study aimed to investigate potential factors associated with the clinical stage of NPC at diagnosis.

**Methods:**

Data were obtained from 118 patients with early-stage NPC and 274 with late-stage NPC who were treated at Sun Yat-sen University Cancer Center between August 2014 and July 2015. Patients were individually matched by age, sex, and residence, and a conditional logistic regression model was applied to assess the associations of clinical stage at diagnosis with socioeconomic status indicators, knowledge of NPC, physical examinations, patient interval, and risk factors for NPC.

**Results:**

Although knowledge of early NPC symptoms, smoking cessation, and patient interval were important factors, the number of cigarettes smoked per day, motorbike ownership, and physical examination exhibited the strongest associations with the clinical stage of NPC at diagnosis. Compared with smoking fewer than ten cigarettes a day, smoking 10–30 cigarettes [odds ratio (OR) 4.03; 95% confidence interval (CI) 1.11–14.68] or more than 30 cigarettes (OR 11.46; 95% CI 1.26–103.91) was associated with an increased risk of late diagnosis. Compared with not owning a motorbike, owning a motorbike (OR 0.38; 95% CI 0.23–0.64) was associated with early diagnosis. Subjects who underwent physical examinations were less likely to receive a late diagnosis than those who did not undergo examinations (OR 0.50; 95% CI 0.28–0.89). However, indicators of wealth were not significant factors.

**Conclusions:**

Initiatives to improve NPC patient prognosis should aim to promote knowledge about early symptoms and detection, health awareness, and accessibility to health facilities among all patients, regardless of socioeconomic status.

## Background

Globally, nasopharyngeal carcinoma (NPC) is a relatively uncommon disease, with estimates of 86,700 new cases and 50,800 deaths worldwide in 2012 [1]. However, NPC is the sixth most common cancer affecting men in South China [1], with incidences as high as 20–30 per 100,000 individuals [2]. Most patients in this region are initially diagnosed with late-stage disease [3, 4]. Among patients with NPC, survival is largely dependent on the clinical stage at diagnosis [5]: the 5-year overall survival rates exceed 90% for early-stage (stage I and II) patients [6, 7], but are less than 50% for late-stage (stage III and IV) patients [8]. Therefore, early diagnosis is essential to improving the prognosis of NPC.

To date, only three studies have investigated the association between the patient interval, defined in accordance with the Aarhus Statement as the time interval between the date of the first symptom and the date of the first presentation (i.e., first medical consultation) which contains “appraisal interval” (time taken to interpret bodily changes/symptoms) and “help-seeking interval” (time taken to act upon those interpretations and seek help) [9], and the diagnosis of late-stage NPC [10–12]. However, these studies neglected to address the interactions among multiple potential factors related to the clinical stage at diagnosis and did not provide information that would facilitate the implementation of measures to improve early NPC diagnostic rates. Therefore, a more comprehensive study of the factors that might affect the clinical stage of NPC at diagnosis is needed.

Existing studies of various cancers have demonstrated a relationship between socioeconomic status (SES) and clinical stage of NPC at diagnosis [13–16]. SES can serve as an indicator of various underlying health-related factors, and individuals with a higher SES may be diagnosed at an earlier stage than those with a lower SES [17–19]. In addition to SES, symptom knowledge [20], physical examinations [21, 22], and patient interval [23] were found to independently associate with the stage of NPC at diagnosis, and factors such as salted fish consumption, cigarette smoking, and a family cancer history are known risk factors for NPC [24, 25]. The knowledge and biological effects of these risk factors might be associated with the clinical stage of NPC at diagnosis and confound the effects of other factors.

Therefore, in the present study, we investigated the associations of the clinical stage of NPC at diagnosis with a series of SES indicators, knowledge of both NPC symptoms and risk factors, physical examinations, patient interval, smoking history, salted fish consumption, and family cancer history among patients at the Sun Yat-sen University Cancer Center (Guangzhou, China), with the aim of developing measures to improve early diagnosis and prognosis of NPC.

## Patients and methods

### Patient selection

We reviewed the clinical records of all NPC patients who were treated between August 1, 2014 and July 31, 2015 at Sun Yat-sen University Cancer Center, the largest cancer hospital in South China. Patients who were diagnosed between 2012 and 2015 and had histologically confirmed NPC were included. Patients with tumors at secondary sites were excluded from this study.

All patients with early-stage (I and II) NPC were selected and interviewed through telephone calls. Subsequently, each patient with early-stage disease was randomly matched with two or three patients with late-stage (III and IV) disease according to residence (urban or rural), age (±5 years), and sex using a random number generation method.

### Data collection

Patients’ characteristics, including sex and tumor stage at diagnosis, were obtained from the hospital electronic record system. Tumor staging was based on the clinical TNM classification in accordance with the 2009 American Joint Committee on Cancer/Union for International Cancer Control (AJCC/UICC) staging system. After providing verbal consent, participants were interviewed via telephone by trained interviewers who used a structured questionnaire that solicited information about SES and other indicators.

SES indicators such as the highest degree of education, occupation, working hours per week, health insurance, residence ownership, residence structure, residence size (m^2^), number of family members living in the residence, and ownership of household appliances were recorded. Occupation was categorized as (i) unskilled workers, including factory laborers, farmers, and other occupations requiring a low degree of education; (ii) skilled workers, including “white-collar” workers, technicians, and other workers with a high degree of education; or (iii) retired or unemployed. Residence ownership was classified as owning or not owning the residence; the latter included rented housing and government- or school-supported housing. Residence structures were categorized into bungalows, houses, apartments, and other types (e.g., dormitory or retail store). Income was shown to be unreliable as a single indicator [26], and was not included in the questionnaire.

We also collected information regarding the participants’ knowledge of early NPC symptoms and risk factors. Early symptoms of NPC include epistaxis, tinnitus and deafness, nasal obstruction, headache, diplopia, and neck masses, whereas risk factors include Epstein–Barr virus (EBV) infection, genetic factors, living environment, and lifestyle [24]. If subjects answered positively to at least one point, they were classified as having knowledge about early symptoms or risk factors of NPC.

We investigated how patients were exposed to the known risk factor for NPC, such as family history of cancer, history of smoking, cigarette consumption, and salted fish consumption [24]. The family history of cancer was classified as no family cancer history, a family history of cancers excluding NPC, or a family cancer history of NPC. The smoking history was categorized as never smoker, former smoker, and current smoker. Cigarette consumption was categorized as fewer than ten cigarettes, 10–30 cigarettes, or more than 30 cigarettes per day. Salted fish consumption was categorized as seldom or never, monthly, or weekly.

Subjects were further divided into two groups by the mean of patient interval (3 months).

### Composite wealth score calculation

To establish a formula for accurate calculation of composite wealth score, we implemented a multiple correspondence analysis (MCA) on the structure, ownership, and area of residence, the ownership of personal car, motorbike, television, computer, air conditioner, vacuum cleaner, and washing machine, and occupation [26]. The MCA was extended with the intent to summarize the associations among a set of categorical variables in a small number of dimensions. In this process, the variables were transformed into new dimensions and given distinct values in each dimension [27]. Variables with more similar values in each dimension are more strongly associated with each other. The first dimension explains as much of the information from the data as possible, whereas each succeeding dimension explains as much of the remaining information as possible. Generally, only the first and second principal dimension scores are used to generate two-dimensional plots, and variables plotted near each other are positively interrelated [28].

A wealth score was computed by evaluating each variable by the weight reported in the first dimension and summing these weights. For example, the first-dimension weights for owning or not owning a car were −1.372 and 1.088, and those for owning or not owning a vacuum cleaner were −1.415 and 0.609. If a subject possessed a car but did not own a vacuum cleaner, the corresponding weights (−1.372 and 0.609) were summed. This process was continued until the weights of all MCA variables were encompassed by this calculation. The composite wealth scores were then computed and used to divide the subjects into tertiles.

### Statistical analysis

We compared the distributions of variables of interest between patients with early-stage and late-stage diseases, using the Chi square test for categorical variables and the Wilcoxon rank-sum test for continuous variables.

In accordance with the matched case–control design of the study, unadjusted and adjusted odds ratios (ORs) and corresponding 95% confidence intervals (CIs) for all study factors were calculated using conditional logistic regression models adjusted for the education level, composite wealth score, and NPC risk factors such as smoking history, salted fish consumption frequency, and family cancer history. When one of these possible confounders was the variable of interest, the other possible confounders were adjusted. All statistical tests were two-tailed, and a *P* value <0.05 was considered significant. Statistical analyses were performed using the R Studio software, version 0.99.893 (https://www.rstudio.com/).

## Results

### Characteristics of patients

Data were obtained from 1488 patients. Among them, only 190 (12.8%) had been diagnosed with early-stage NPC, and related information was successfully collected from 118 of these patients. Of the 118 early-stage patients, 80 were each matched with two patients with late-stage disease, and 38 were each matched with three patients with late-stage disease; therefore, a total of 274 late-stage patients were recruited. Overall, 66.3% of patients were male (77 with early-stage disease and 183 with late-stage disease), and 33.7% were female (41 with early-stage disease and 91 with late-stage disease). Furthermore, 71.4% of patients (83 with early-stage disease and 197 with late-stage disease) resided in urban regions, and 28.6% (35 with early-stage disease and 77 with late-stage disease) in rural regions. The median age was 43 years for both early-stage and late-stage patients, with ranges of 16–68 years and 14–72 years, respectively. Because of the matched design, no differences were observed between the two groups.

### MCA of SES indicators

**Fig. 1:**
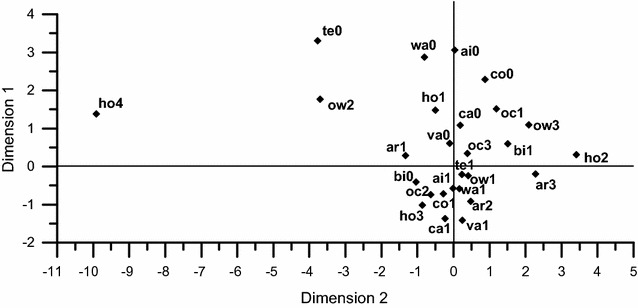
Visualization of the coordinates of wealth variables included in the multiple correspondence analysis (MCA) of 118 early-stage nasopharyngeal carcinoma (NPC) patients and 274 late-stage NPC patients. Appliance ownership: *bi* motorbike, *co* computer, *va* vacuum cleaner, *wa* washing machine, *ca* automobile, *ai* air conditioner, *te* television. The suffix “*0*” indicates non-ownership of the appliance; the suffix “*1*” indicates ownership. Housing: *ow1* owned a residence, *ow2* rented a residence, *ow3* other types of ownership, *ho1* bungalow, *ho2* house, *ho3* apartment, *ho4* other types of residence structure, *ar1* first tertile of residence area, *ar2* second tertile of residence area, *ar3* third tertile of residence area. Occupation: *oc1* unskilled workers, *oc2* skilled worker, *oc3* retired or unemployed. The indicators associated with higher wealth gather along the *bottom half* of the plot, whereas the indicators of lower wealth are located on *the upper half*. Dimensions 1 and 2 cover 76.7% and 10.2% of the total Chi square variations in the data

Figure 1 displays the results for the first two dimensions (axes) of the MCA, which included all patients with early-stage and late-stage diseases. Because the first dimension covered most (76.7%) of the total Chi square variations in the data, it was used for further analyses. In this figure, the indicators associated with higher wealth are presented at the bottom half of the plot, whereas the indicators of lower wealth are located on the upper half.

### Association between SES and NPC stage at diagnosis

The ORs (95% CIs) for the associations of SES indicators with clinical stage of NPC at diagnosis are displayed in Table 1. Compared with patients who had a middle school or lower education level, patients with a high school or higher education level was not associated with a decreased risk of late diagnosis (adjusted OR 0.58; 95% CI 0.32–1.05). Before model adjustment, skilled workers were more likely than unskilled workers to receive an early diagnosis (crude OR 0.52; 95% CI 0.28–0.98), but this association disappeared after model adjustment (adjusted OR 0.57; 95% CI 0.27–1.22). Neither health insurance nor working hours per week was associated with the risk of late diagnosis. Those lived with 2–3 family members were more likely to receive an early diagnosis than those lived with 0–1 family members (adjusted OR 0.48; 95% CI 0.23–0.98). After model adjustment, no associations of residence ownership, residence structure, residence size, and residence size per person were observed with the risk of late diagnosis. Interestingly, owning a motorbike (OR 0.38; 95% CI 0.23–0.64) was associated with an early diagnosis after adjusting for education level, smoking history, salted fish consumption, and family cancer history, except for the wealth score. This association remained after the wealth score was added to the adjusted factors (OR 0.29; 95% CI 0.16–0.41). No associations were observed for the ownership of other appliances.

**Table 1:** Associations of potential factors with NPC stage at diagnosis

Variable	NPC stage at diagnosis [cases (%)]		Crude OR (95% CI)	Adjusted OR (95% CI)^a^
	Early stage	Late stage		
Education level				
Middle school or lower	45 (38.1)	132 (48.2)	1.00	1.00
High school or higher	73 (61.9)	142 (51.8)	0.56 (0.33–0.95)*	0.58 (0.32–1.05)
Occupation				
Unskilled workers	22 (18.6)	67 (24.5)	1.00	1.00
Skilled workers	75 (63.6)	144 (52.5)	0.52 (0.28–0.98)*	0.57 (0.27–1.22)
Retired or unemployed	21 (17.8)	63 (23.0)	0.95 (0.44–2.06)	0.99 (0.42–2.32)
Working hours per week^b^				
0–30	33 (28.2)	87 (31.8)	1.00	1.00
31–50	48 (41.0)	91 (33.2)	0.70 (0.38–1.28)	0.75 (0.38–1.48)
51 or more	36 (30.8)	96 (35.0)	0.95 (0.50–1.78)	0.81 (0.40–1.63)
Insurance				
UBMI1	72 (61.0)	168 (61.3)	1.00	1.00
NRCMS	37 (31.4)	86 (31.4)	1.13 (0.58–2.22)	1.14 (0.50–2.60)
UBMI2	6 (5.1)	4 (1.5)	0.25 (0.07–0.91)*	0.31 (0.08–1.23)
Others	3 (2.5)	16 (5.8)	2.60 (0.72–9.41)	2.10 (0.51–8.62)
Number of family members^c^				
First tertile (1–2)	12 (10.3)	52 (19.0)	1.00	1.00
Second tertile (3–4)	75 (64.7)	129 (47.1)	0.41 (0.21–0.82)*	0.48 (0.23–0.98)*
Third tertile (≥5)	29 (25.0)	93 (33.9)	0.73 (0.34–1.55)	0.83 (0.37–1.86)
Residence ownership^d^				
No	18 (15.4)	31 (11.3)	1.00	1.00
Yes	99 (84.6)	243 (88.7)	1.35 (0.73–2.49)	1.77 (0.81–3.88)
Residence structure				
Bungalow	31 (26.3)	88 (32.1)	1.00	1.00
House	22 (18.6)	51 (18.6)	0.82 (0.43–1.55)	1.04 (0.51–2.12)
Apartment	64 (54.2)	133 (48.6)	0.68 (0.40–1.17)	0.99 (0.50–1.96)
Others	1 (0.8)	2 (0.7)	0.69 (0.06–8.04)	0.68 (0.06–8.17)
Residence size (m^2^)				
First tertile (<100)	66 (56.0)	160 (58.4)	1.00	1.00
Second tertile (100–120)	19 (16.0)	24 (8.8)	0.52 (0.27–1.02)	0.61 (0.29–1.29)
Third tertile (>120)	33 (28.0)	90 (32.8)	1.15 (0.70–1.90)	1.13 (0.65–1.96)
Area per person (m^2^)^e^				
First tertile (<24)	27 (23.3)	82 (30.0)	1.00	1.00
Second tertile (24–35)	50 (43.1)	96 (35.0)	0.60 (0.33–1.07)	0.64 (0.33–1.24)
Third tertile (>35)	39 (33.6)	96 (35.0)	0.76 (0.42–1.38)	0.80 (0.41–1.55)
Appliance ownership^f^				
Car	56 (48.7)	116 (42.3)	0.74 (0.46–1.20)	0.78 (0.47–1.29)
Motorbike	63 (54.3)	97 (35.4)	0.45 (0.29–0.71)*	0.38 (0.23–0.64)*
Computer	89 (76.7)	207 (75.5)	0.84 (0.46–1.53)	0.86 (0.45–1.63)
Television	111 (95.7)	256 (93.4)	0.58 (0.20–1.66)	0.66 (0.22–2.03)
Air conditioner	102 (87.9)	226 (82.5)	0.48 (0.22–1.05)	0.52 (0.23–1.19)
Washing machine	97 (83.6)	227 (82.8)	0.86 (0.44–1.66)	0.93 (0.46–1.87)
Vacuum cleaner	38 (32.8)	80 (29.2)	0.86 (0.54–1.36)	0.89 (0.54–1.45)
Wealth score^g^				
First tertile (<−3.9)	44 (37.3)	83 (30.3)	1.00	1.00
Second tertile (−3.9 to 1.1)	38 (32.2)	93 (33.9)	1.29 (0.76–2.19)	1.26 (0.72–2.20)
Third tertile (>1.1)	36 (30.5)	98 (35.8)	1.64 (0.90–2.99)	1.75 (0.88–3.47)
Family cancer history				
None	78 (66.1)	189 (69.0)	1.00	1.00
NPC	18 (15.3)	36 (13.1)	0.86 (0.46–1.61)	0.78 (0.40–1.53)
Other cancers	22 (18.6)	49 (17.9)	0.95 (0.54–1.67)	0.93 (0.51–1.72)
Smoking history				
Never smoker	74 (62.7)	160 (58.4)	1.00	1.00
Former smoker	18 (15.3)	14 (5.1)	0.35 (0.14–0.85)*	0.29 (0.11–0.77)*
Current smoker	26 (22.0)	100 (36.5)	1.72 (0.93–3.19)	1.75 (0.92–3.32)
Cigarette number per day^h^				
<10	22 (51.2)	31 (27.2)	1.00	1.00
10–30	19 (44.2)	66 (57.9)	2.87 (1.14–7.25)*	4.03 (1.11–14.68)*
>30	2 (4.6)	17 (14.9)	6.04 (1.09–33.32)*	11.46 (1.26–103.91)*
Salted fish consumption^i^				
Seldom or never	72 (61.5)	131 (48.0)	1.00	1.00
Every month	25 (21.4)	70 (25.6)	1.58 (0.91–2.74)	1.53 (0.85–2.73)
Every week	20 (17.1)	72 (26.4)	1.89 (1.07–3.34)*	1.71 (0.93–3.17)
Knowledge of NPC early symptoms				
No	40 (33.9)	138 (50.4)	1.00	1.00
Yes	78 (66.1)	136 (49.6)	0.54 (0.35–0.83)*	0.60 (0.37–0.98)*
Knowledge of NPC risk factors				
No	88 (74.6)	223 (81.4)	1.00	1.00
Yes	30 (25.4)	51 (18.6)	0.66 (0.38–1.12)	0.74 (0.42–1.31)
Physical examination				
Never	51 (43.2)	167 (60.9)	1.00	1.00
Ever	67 (56.8)	107 (39.1)	0.40 (0.24–0.67)*	0.50 (0.28–0.89)*
Patient interval (months)				
≤3	95 (80.5)	188 (68.6)	1.00	1.00
>3	23 (19.5)	86 (31.4)	1.87 (1.13–3.11)*	1.83 (1.07–3.13)*

*OR* odds ratio, *CI* confidence interval, *UBMI1* urban basic medical insurance uncovering state-owned enterprise employees and government staffs, *NRCMS* new rural cooperative medical scheme, *UBMI2* urban basic medical insurance of state-owned enterprise employees and government staffs

* Statistically significant results

^a^Adjusted for education level, wealth score, smoking history, salted fish consumption, and family cancer history. When one of these possible confounders was the variable of interest, the other possible confounders were adjusted

^b^Data was missing for one patient in the early stage group

^c^Data was missing for two patients in the early stage group

^d^Data was missing for one patient in the early stage group

^e^Data was missing for two patients in the early stage group

^f^For each appliance, the reference group comprised the subjects that did not own the appliance. The multivariate analysis of these appliance ownership variables was not adjusted for the wealth score

^g^Because appliances associated with higher wealth are located on the bottom half of the multiple correspondence analysis (MCA) graph, a lower score indicates a higher level of wealth

^h^We only included former and current smokers. The multivariate analysis of the number of cigarettes per day was not adjusted for the smoking history

^i^Data was missing for one patient in the early stage group and one in the late stage group

For the results of composite wealth score, we have to note that the wealthiest patients had negative scores because expensive appliances were located on the bottom half of the MCA plot. Compared with a composite wealth score lower than −3.9, a composite wealth score higher than 1.1 showed no significant association with late diagnosis of NPC in either the univariate or the multivariate models, although the point estimate was quite bigger than one.

### Association between NPC risk factors and NPC stage at diagnosis

As shown in Table 1, a family cancer history was not found to associate with the risk of late diagnosis. Surprisingly, smoking cessation, compared with no smoking history, was associated with early diagnosis (adjusted OR 0.29; 95% CI 0.11–0.77). Smoking more than 30 (adjusted OR 11.46; 95% CI 1.26–103.91) or 10–30 cigarettes per day (adjusted OR 4.03; 95% CI 1.11–14.68) was significantly associated with the risk of a late diagnosis when compared with smoking fewer than ten cigarettes per day (*P* for trend = 0.015). Compared with seldom or never eating salted fish, eating salted fish every week was associated with an increased risk of late diagnosis in the univariate analysis (crude OR 1.89; 95% CI 1.07–3.34), but not in the multivariate analyses (adjusted OR 1.71; 95% CI 0.93–3.17).

### Associations between physical examination, patient interval, NPC-related knowledge, and NPC stage at diagnosis

As shown in Table 1, knowing about NPC early symptoms was associated with an increased likelihood of early diagnosis as compared with not having the knowledge (adjusted OR 0.60; 95% CI 0.37–0.98). In contrast, no association was observed between knowledge about risk factors and an early diagnosis. Taking physical examination increased the likelihood of an early diagnosis (adjusted OR 0.50; 95% CI 0.28–0.89). In addition, a patient interval of 3 or more months was associated with an increased risk of late diagnosis (adjusted OR 1.83; 95% CI 1.07–3.13), compared with a shorter interval.

## Discussion

The present study identified associations of several SES indicators, including the number of family members living together and whether owning a motorbike, with the clinical stage of NPC at diagnosis. However, the composite wealth score and other SES indicators were not found to associate with the risk of late diagnosis. In addition, knowledge about early symptoms of NPC, the patient interval, physical examination, and smoking history and frequency were found to associate with the clinical stage of NPC at diagnosis.

It can be difficult to collect income-related information during epidemiological studies, and wealth can differ dramatically across different social groups even if incomes are similar [29]. Investigations of nonmonetary indicators of wealth, such as appliance ownership, housing conditions, and occupation, have been shown to be less sensitive and relatively accurate [26, 30]. We therefore collected information about the patients’ occupations, residence ownership, size, and structure, and household appliance ownership to measure their wealth. Interestingly, although owning a car or vacuum cleaner was associated with the highest level of wealth in the MCA, they were not related to an early diagnosis of NPC. In contrast, owning a motorbike was associated with an early diagnosis after adjusting for the wealth score, suggesting that this factor might substitute for other factors in the exclusion of wealth. In the present study, patients in rural areas were statistically more likely to own a motorbike than those in urban areas (data not shown). In addition, as many Chinese cities have banned motorbikes in downtown areas [31], most urban motorbike owners live in suburbs. Motorbikes provide patients in rural and suburban areas with a rapid, convenient transportation to healthcare facilities, which tend to be located in relatively distant areas [14]. We speculated that owning a car, an indicator of wealth, was a characteristic of subjects who lived in downtown areas with adjacent healthcare facilities that were also easy to reach by public transportation [14]. Therefore, car ownership might not provide the same significant benefit as motorbike ownership.

The association between a high education level and early NPC diagnosis was not significant in our study. In contrast, the association between knowledge about early NPC symptoms and early diagnosis was significant. Therefore, it is reasonable to assume that knowledge about early symptoms had a stronger effect on the likelihood of an early diagnosis of NPC than the general education level. Accordingly, we recommend that governmental and healthcare facilities educate the public about early NPC symptoms to effectively increase the early diagnosis rate.

We surveyed several factors associated with living conditions and household density, including the total residence size, number of family members living together, and residence size per person, as potential indicators of SES. A shared residence with 3–4 family members, compared with 1–2 family members, was more strongly associated with an early diagnosis of NPC, which could be attributed to the increased support and intimate relationships among family members [32]. For example, close family relationships might lead to the disclosure of a symptom to a family member and could potentially reduce the delay in presenting the symptom to a general practitioner [33]. However, people living in crowded accommodations might also be prone to insufficient diets and infectious disease [34, 35], which could explain why living with 5 or more family members was not related to the likelihood of an early diagnosis.

We used a composite wealth score, calculated by adding the MCA scores for ownership of different appliances, occupation, and residence structure, size, and ownership, to measure each patient’s overall wealth status. This measurement of wealth was proved to be accurate in another study conducted in an under-developed country [26]. In the present study, we did not find an association of the composite wealth score or any other wealth indicators with the clinical stage at diagnosis, leading us to postulate that wealth is not associated with the clinical stage of NPC at diagnosis. The composite wealth score was further used as an integrated variable to control the potential confounding bias induced by the components of the score.

A patient interval of 3 months or longer was associated with a late diagnosis of NPC, which agreed with the findings of similar studies in which physical examination and knowledge about early symptoms played important roles in reducing the patient interval and promoting diagnosis at an early stage [36, 37]. In the present study, physical examination was also associated with early NPC diagnosis. The examinations tend to include cancer screening tests and are considered useful for reducing the patient interval and detecting cancer at early stages [22, 38]. For NPC, EBV serology test was found to be a useful NPC screening tool in a high-risk population [39, 40]. Similarly, knowledge about early symptoms of NPC was associated with early diagnosis, consistent with previous studies that described the recognition and perception of symptoms as the first and most important step toward seeking medical help and reducing the patient interval [41–44]. On the other hand, no significant association was found between knowledge about NPC risk factors and early diagnosis of NPC, in contrast to the findings of a study on oral and pharyngeal carcinoma [44]. However, known NPC risk factors, such as cigarette smoking history and frequency, were associated with late diagnosis of NPC in the present study, suggesting that knowledge about risk factors did not lead to positive health behaviors. Therefore, to reduce the patient interval and increase the rate of early diagnosis, promoting physical examinations and education about early symptoms of NPC could be more effective than promoting education about NPC risk factors.

In the present study, smoking cessation was associated with early NPC diagnosis when compared with never smoked. We assumed that people who had quit smoking may have been more conscious of health issues. In previous studies, most smokers expressed regret that they had begun smoking and worried about the future effects of smoking on their health and quality of life [45, 46]. Furthermore, smokers who had quit or intended to quit smoking were likely to have more access to beneficial health information [47]. However, the pernicious effects of smoking are undisputed. We found that daily smoking amount was strongly associated with late diagnosis of NPC in a dose–response manner indicated by the observed significant trends. We further assumed that smoking is a significant tumor growth promoting factor, because cigarette smoke has mutagenic and DNA-damaging effects that lead to the malignant transformation of normal epithelial cells in the nasopharynx [48, 49]. Immunologically, cigarette smoking may suppress natural killer cell activation and cytotoxicity, which could further alter the tumor microenvironment, promote tumor progression and metastasis, and increase the tumor burden [50, 51].

In the present study, we used a case–control design to establish a new, efficient approach so as to identify potential factors associated with the clinical stage of NPC at diagnosis. In case–control studies, the cases are usually patients who have experienced relatively rare events. In the present study, only 190 of 1488 NPC patients were diagnosed at an early stage. Therefore, we defined early-stage NPC patients as cases and matched them with late-stage NPC patients by sex, age, and residency. This design allowed us to find subtler differences between late-stage and early-stage NPC patients. If we had designated late-stage NPC patients as cases, we might not have had an adequate number of matched early-stage NPC patients. We note that as few patients are diagnosed at early stages in other cancers, this approach could be implemented in similar studies.

The present study probed potential factors associated with the clinical stage of NPC at diagnosis by investigating a comprehensive number of possible factors with individual-level indicators. Unlike similar studies of other cancers that used neighborhood-level indicators [14–16], individual-level indicators are more accurate and less prone to ecological bias and misclassification. Moreover, the use of the composite wealth score rather than a single indicator allowed us to more accurately measure patients’ wealth statuses [26]. To eliminate confounding effect between indicators, we adjusted for several important potential confounders, such as NPC risk factors, the composite wealth score, and education. Although many factors were statistically insignificant, the indicators with null associations would at least have weaker effects on clinical NPC stage at diagnosis than the indicators that were significant.

Because the interactions among viral infections, genetic factors, and environment are not fully understood, secondary NPC prevention tends to be more cost-effective than primary prevention [2]. Measures aimed at improving the early NPC diagnosis rate will likely increase survival rates and ease the medical burdens on patients and healthcare systems. Our findings suggest that high-risk populations should be targeted to improve health awareness by providing education related to early NPC symptoms and encouraging routine physical examinations. Furthermore, governments should provide more convenient access to healthcare facilities, and smokers should reduce and quit smoking as soon as possible. Contrary to other studies, we found that measures such as improving the general education level and SES might not be as effective as the measures mentioned above. However, the present study only provides a general orientation, and we hope future studies will offer more detailed measures.

One limitation of this study was the collection of information about previous exposures. Nevertheless, we reduced the likelihood of recall bias by surveying facts that could be recalled truthfully, such as the level of education or occupation. Another limitation was the relatively small number of study subjects and inclusion of patients from a single hospital. Accordingly, we could not exclude an occasional association. A multicenter study with a large sample is needed to confirm our results.

## Conclusions

In summary, the present study suggested that factors related to cigarette smoking, easy access to healthcare facilities, health awareness, knowledge of early NPC symptoms, and early NPC detection were strongly associated with the clinical stage of NPC at diagnosis. In contrast, the wealth score and other SES indicators had limited effects. Therefore, to increase the rate of early diagnosis and improve the prognosis of NPC patients, high-risk populations should be targeted by initiatives aimed at improving health awareness and reducing patient interval through education on early NPC symptoms, promoting routine physical examination, and improving the accessibility to healthcare facilities. Furthermore, smokers must reduce their cigarette consumption and ultimately quit smoking. However, multicenter large-sample studies are needed to confirm our results. Finally, the case–control design of the present study provided a new, efficient approach to the investigation of factors related to the clinical stage of NPC at diagnosis.

## Abbreviations

NPC: nasopharyngeal carcinoma
SES: socioeconomic status
MCA: multiple correspondence analysis
AJCC/UICC: American Joint Committee on Cancer/Union for International Cancer Control
UBMI1: urban basic medical insurance excluding state-owned enterprise employees and government staffs
NRCMS: new rural cooperative medical scheme
UBMI2: urban basic medical insurance of state-owned enterprise employees and government staffs
